# Arachidonic and docosahexaenoic acid supplementation affects white matter microstructure in preterm infants

**DOI:** 10.1093/braincomms/fcag362

**Published:** 2026-10-11

**Authors:** Gunnthorunn Gunnarsdottir, Ivan I Maximov, Oliver Marcel Geier, Tone Nordvik, Anette Ramm-Pettersen, Tom Stiris, Sissel J Moltu, Helle Cecilie Viekilde Pfeiffer

**Affiliations:** Department of Pediatric Neurology, Oslo University Hospital, 0424 Oslo, Norway; Institute of Clinical Medicine, University of Oslo, 0316 Oslo, Norway; Department of Health and Functioning, Western Norway University of Applied Sciences, 5025 Bergen, Norway; Department of Physics and Computational Radiology, Division of Radiology and Nuclear Medicine, Oslo University Hospital, 0372 Oslo, Norway; Department of Neonatal Intensive Care, Oslo University Hospital, Oslo 0424, Norway; Department of Pediatric Neurology, Oslo University Hospital, 0424 Oslo, Norway; Institute of Clinical Medicine, University of Oslo, 0316 Oslo, Norway; Institute of Clinical Medicine, University of Oslo, 0316 Oslo, Norway; Department of Neonatal Intensive Care, Oslo University Hospital, Oslo 0424, Norway; Department of Neonatal Intensive Care, Oslo University Hospital, Oslo 0424, Norway; Paediatric and Neurological Departments, Danish Epilepsy Centre, 4293 Dianalund, Denmark

**Keywords:** preterm infants, fatty acids, advanced diffusion MRI, white matter

## Abstract

Arachidonic acid and docosahexaenoic acid are long-chain polyunsaturated fatty acids that serve as key structural components of neuronal cell membranes and exhibit anti-inflammatory properties. They accumulate most rapidly during the third trimester of pregnancy, leaving very preterm infants deprived at birth. We conducted a double-blind randomized controlled trial comparing arachidonic and docosahexaenoic acid supplementation to medium chain triglycerides oil and assessed effects on white matter maturation using advanced diffusion imaging techniques. We used tract-based spatial statistics to localize significant changes across major tracts. Diffusion kurtosis imaging and Bayesian rotation invariant approach were applied to describe white matter microstructure within the intra- and extra-axonal water compartments enabling a more detailed characterization of tissue changes. These advanced diffusion MRI data were acquired at term-equivalent age for 64 infants born before 29 weeks of gestation, including 33 in the supplemented group and 31 in the control group. We identified significantly higher fractional anisotropy and lower radial diffusivity in several white matter regions in the supplemented group, mostly pronounced in areas that myelinate early. There were no significant differences in diffusion kurtosis imaging metrics between the two groups, but the Bayesian rotational invariant approach revealed several significant differences. This might illustrate a different developmental trajectory in the supplemented group compared to the control group. Applying advanced diffusion models can potentially provide a deeper understanding of early brain development and facilitate opportunities for early detection and intervention for preterm children.

## Teaser

Gunnarsdottir *et al.* report that supplementing preterm infants with arachidonic and docosahexaenoic acid promotes early white matter maturation. Advanced diffusion magnetic resonance imaging indicates a different developmental trajectory and potential for earlier detection and intervention.

## Graphical Abstract

**Graphical Abstract:**
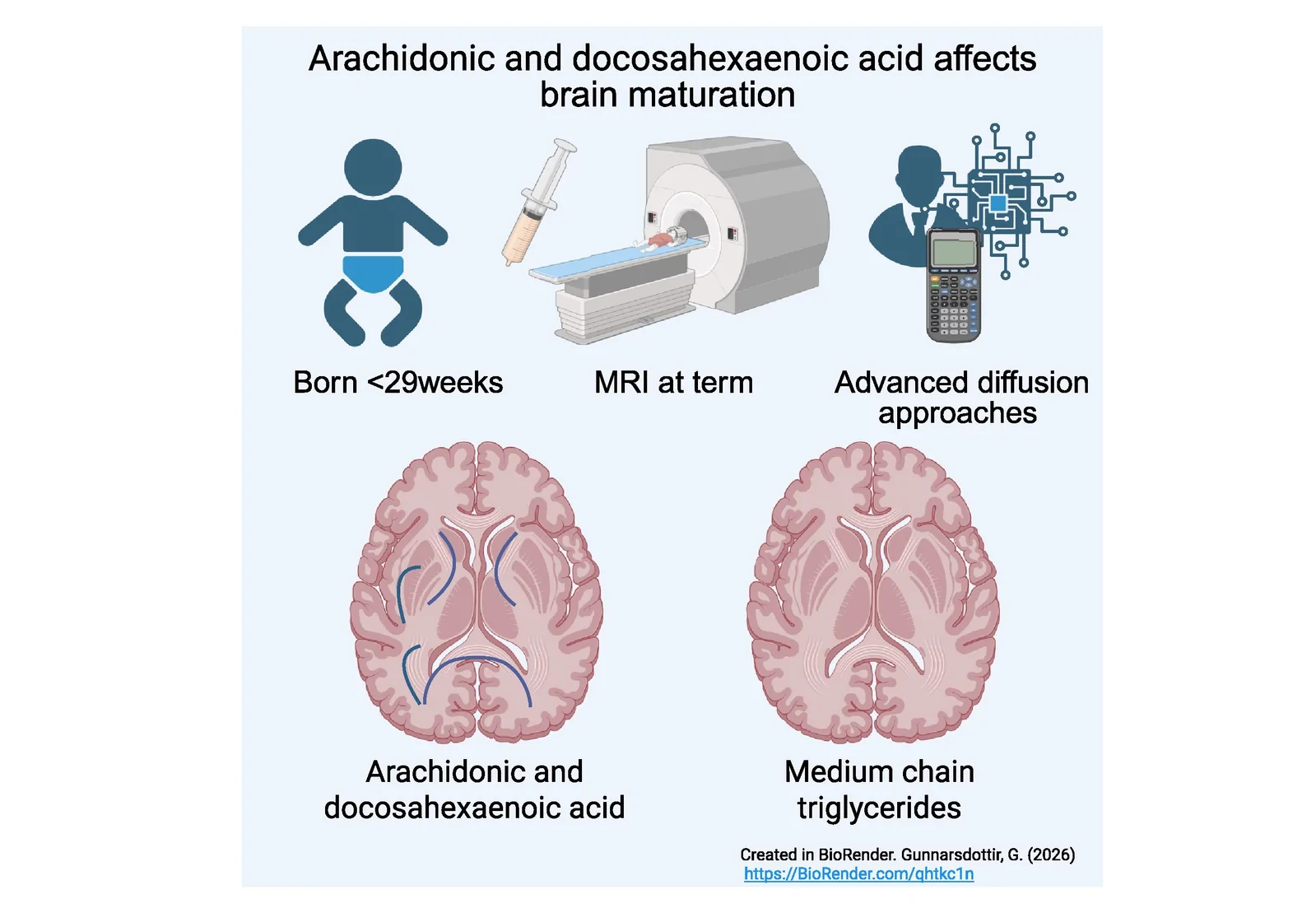

## Introduction

The final trimester of pregnancy is critical for brain development during which neuronal migration, dendritic arborization, sulci formation and synaptogenesis occur.^1^ The mechanism underlying altered white matter (WM) maturation^2^ in very preterm infants are multifactorial, including post-natal malnutrition^1^ and sustained inflammation.^3^

Arachidonic acid (ARA) and docosahexaenoic acid (DHA) are long-chain polyunsaturated fatty acids abundant in the cell membranes in the brain and critical for neurodevelopment.^4^ Very preterm infants are deprived of the high placental transfer of ARA and DHA in the third trimester.^5^ Thus, supplementation of these fatty acids may improve WM maturation.^6,7^

We have previously reported a potential protective effect of ARA:DHA supplementation on WM development in preterm infants assessed with diffusion tensor imaging (DTI) MRI at term-equivalent age.^8^ We found that the ARA:DHA group displayed significantly higher fractional anisotropy (FA) and lower radial diffusivity (RD) in several WM tracts compared to the control group,^8^ suggesting better axonal organization and myelination.

Despite its foundational role in clinical neuroimaging, conventional DTI lacks the specificity needed to disentangle WM tissue composition into its underlying microstructural constituents, such as axonal fibres, cellular structures and diffusivities of extra-axonal space. DTI likely oversimplifies the diffusion in biological tissue and limits the ability to visualize microstructural changes in WM, especially in regions with crossing fibres or dense cellular environments.^9^ To address these limitations, the present study applied more advanced models, specifically diffusional kurtosis imaging (DKI) and Bayesian rotation invariant approach (BRIA). These models may offer improved sensitivity and specificity by capturing heterogeneity and compartmental diffusion characteristics in more detail.^10,11^ DKI quantifies deviations from Gaussian water diffusion that arise from the complex microstructural organization of neural tissue.^11^ In turn, BRIA enables a biologically informed decomposition of the underlying WM architecture into intra- and extra-axonal compartments, allowing compartment-specific estimation of water diffusivity that reflects axonal coherence and extracellular microenvironment. Previous large-scale BRIA studies in adult populations have shown strong age sensitivity and utility in identifying vulnerable tracts related to neurodegenerative conditions, like Alzheimer’s disease.^12,13^ These studies displayed age-related decrease in intra-axonal water fraction with increasing age, increasing extra-axonal and free water fraction and decreasing kurtosis metrics; however, studies in preterm infants are limited. Accordingly, the aim of this study was to characterize the microstructural effects of early ARA and DHA supplementation on WM maturation in very preterm infants by applying advanced diffusion approaches, specifically DKI and BRIA, to a subgroup with high-quality diffusion MRI. We hypothesized that advanced diffusion approaches would detect compartment-specific microstructural effects of ARA and DHA supplementation that were not captured by conventional DTI.

## Materials and methods

### Study design and participants

We used a subset of the full ImNuT cohort^14^ that originally consisted of 120 preterm infants born with a gestational age (GA) less than 29 weeks at Oslo University Hospital without major congenital malformations, chromosomal abnormalities or critical illness with short life expectancy (see Fig. 1). The study was registered at ClinicalTrials.gov (NCT03555019), approved by the Regional Committee for Medical and Health Research Ethics (2016-003700-31) and reviewed for safety and efficacy by a data monitoring committee who also performed a safety interim analysis. The principles of the Consolidated Standards of Reporting Trials (CONSORT) guideline were followed. Written informed consent was obtained from both parents within 48 h after birth. The consent form and other parental information was made in collaboration with a representative from the Norwegian Society of Premature Birth. Patients were allocated to one of two treatment groups using a computer-generated random number list. Block sizes of four, six and eight were used, with a 1:1 allocation within the block. One group received 100 mg/kg of ARA and 50 mg/kg of DHA (Formulaid™, DSM Nutritional Products Inc.) and the other medium chain triglycerides (MCT-oil™, Nutricia) as a daily enteral bolus. Supplementation was started on the second day of life and gradually increased and administered until 36 weeks post menstrual age.^14^ MCT-oil is currently the standard lipid supplement in our department to support enteral energy intake, although typically given at higher doses than those used in this study. In addition to the enteral fatty acid supplement, all study participants followed a previously published nutritional protocol.^14,15^ Nutrient intakes were assessed using a computer-aided nutrition calculation programme (Nutrium software, Nutrium AB, Umeå, Sweden). Participants with poor image quality or who were unable to complete the full MRI protocol were not included in this analysis.

**Figure 1:**
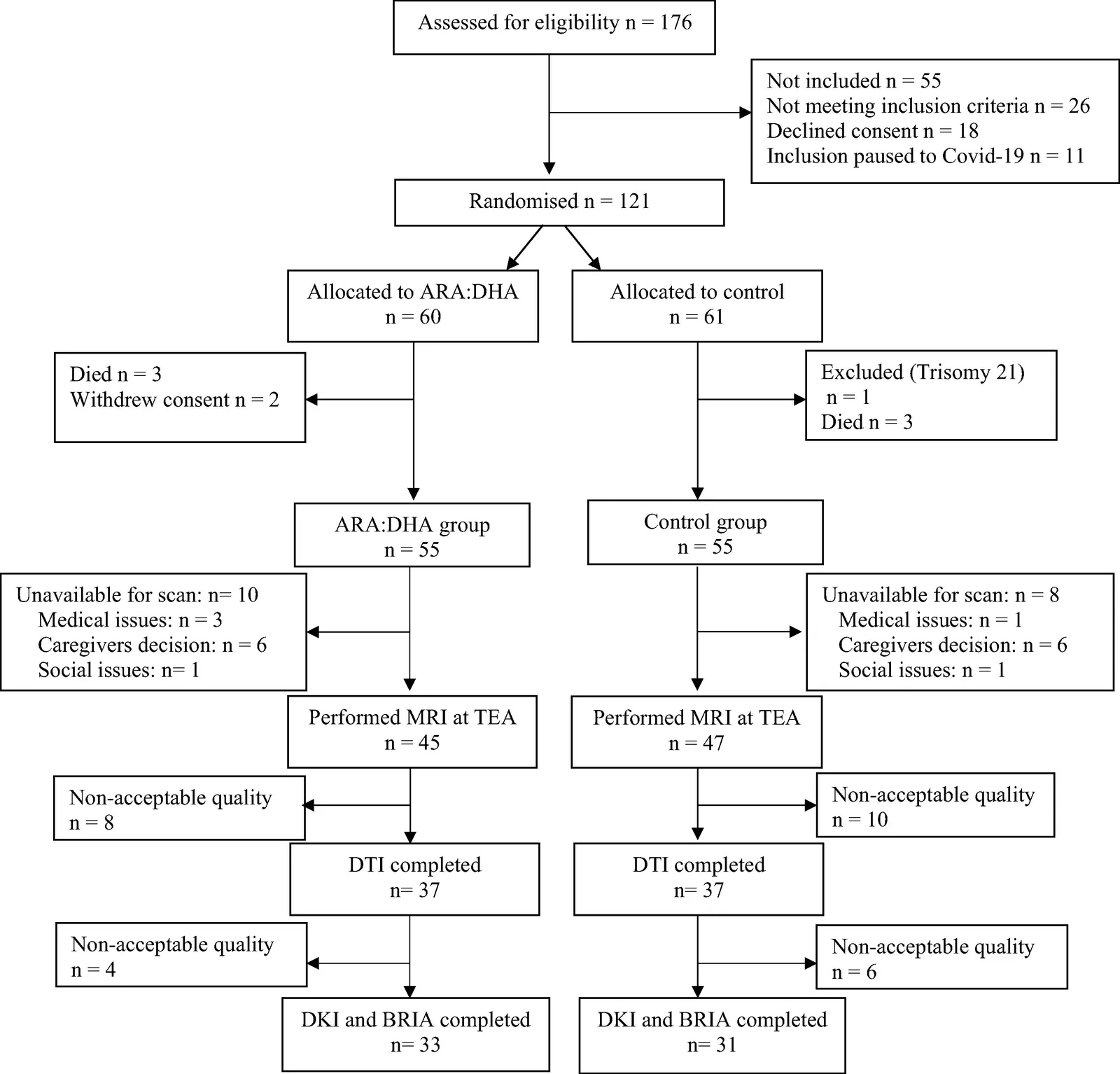
**Flowchart of study participants in the ImNuT trial**. ARA, arachidonic acid; DHA, docosahexaenoic acid; TEA, term-equivalent age; DTI, diffusion tensor imaging; DKI, diffusional kurtosis imaging; BRIA, Bayesian rotation invariant approach.

### MRI acquisition

Diffusion measurements were performed using a 3T Siemens Magnetom Prisma MRI scanner with a 32-channel head coil (Siemens Healthineers, Erlangen, Germany).

MRI scans were conducted at term-equivalent age during natural sleep using the ‘Feed-and-swaddle’ technique (Baby-fix Cocoon, Pearl Technology AG, Schlieren, Switzerland). For acoustic noise protection, we used dental putty (Provil Novo putty soft fast, Jacobsen A/S, Oslo Norway) and soft cushions. Infants were monitored with a 3T compatible pulse-oximeter (Tesla dUO, MIPM GmbH, Mammendorf, Germany) and a video camera (Model 12M−I, MRC Systems GmbH, Heidelberg, Germany).

The raw diffusion-weighted imaging data were acquired using a single-shot spin-echo multiband sequence with a monopolar gradient scheme.^16^ The diffusion protocol allowed for whole brain coverage with an isotropic resolution of 1.5 × 1.5 × 1.5 mm^3^. Multiband acceleration factor was 4 and TE/TR = 84 ms/3.2 s. Diffusion-weighted data had three *b*-values equal to 200 (10 directions), 1000 (30 directions) and 2000 (combined acquisition with 24 + 26 directions) s/mm^2^. For susceptibility distortion correction, additional *b* = 0 images were acquired with opposite phase encoding direction.

### MR data analysis

The raw diffusion data were analysed using an optimized post-processing pipeline.^17^ The pipeline consists of the following steps: noise correction,^18^ Gibbs ringing correction,^19^ correction of susceptibility, eddy-current and motion artefacts using a combination of topup^20^ and eddy^21^ utilities from FSL package (version 6.0.1).^22^ The processed raw data were smoothed using 0.85 mm^3^ Gaussian kernel by fslmaths utility from the FSL package.

We calculated DTI and DKI diffusion scalar metrics based on the diffusion kurtosis approach expanding the diffusion signal.^23^ These scalar maps provide insights into general tissue integrity by probing its macroscopic changes including significant tissue anisotropy inside the voxel or variation in RD as an indirect proxy of WT myelination. For further details on the methodology, refer to Box 1 and Fig. 2.

Box 1 Description of the used diffusion approaches and derived scalar maps

**Table:** 

	Diffusion approach	Derived diffusion metrics
Diffusion tensor imaging (DTI)	Analysis of the degree and direction of water diffusion within each voxel^24^. The diffusion tensor is estimated from the measurement of a diffusion coefficient along multiple directions. The method is based on a Gaussian approximation that water diffuses evenly^11^. Commonly used for reconstructing WM-fibre tracts.	Fractional anisotropy (FA): the directional preference of diffusion where 0 indicates isotropic diffusion and 1 indicates anisotropic diffusion Mean diffusivity (MD): average of diffusion in all directions Radial diffusivity (RD): diffusion in directions across axons Axial diffusivity (AD): diffusion along the length of axons
Diffusional kurtosis imaging (DKI)	An extension of DTI that assesses non-Gaussian diffusion through the estimation of diffusional kurtosis. Kurtosis quantifies how much the actual diffusion deviates from the Gaussian assumption^11^. DKI metrics are sensitive to the complex tissue organization.	Mean kurtosis: overall deviation of diffusion from Gaussian distribution across all directions Axial kurtosis: deviation of diffusion along the main axis of the WM tracts Radial kurtosis: deviation of diffusion perpendicular to the main axis of WM tracts (across axonal fibres)
Bayesian rotation invariant approach (BRIA)	BRIA is an extensive diffusion model that distinguishes between three different water pools within WM: intra-axonal space, extra-axonal space and partial volume water (free water). The parameters are fitted using Bayes theorem. Distributions of the diffusion coefficients were based on updated reference numbers^25^. Microscopic FA, MD, AD and RD are similar to the conventional DTI's parameters, except they are estimated from the combination of intra- and extra-axonal diffusion coefficients. The combination of scalar intra- and extra-axonal metrics, along with rotational invariance, allows for a more detailed and less orientation-dependent characterization of white matter microstructure	Intra-axonal axial diffusivity (D_IAW_): diffusivity inside and along axons. Perpendicular intra-axonal diffusivity is negligible Extra-axonal axial diffusivity (D_EAW_): diffusivity along axons in extra-axonal space Extra-axonal radial diffusivity (D_ERW_): diffusivity across axons in extra-axonal space Microscopic FA (microFA) Microscopic MD (microMD) Microscopic AD (microAD) Microscopic RD (microRD) Intra-axonal water fraction (V_IAW_): portion of water inside axons Extra-axonal water fraction (V_EAW_): portion of water around axons Free-water fraction (V_CSF_): portion of water in the voxel with isotropic diffusion (like CSF or oedema)

**Figure 2:**
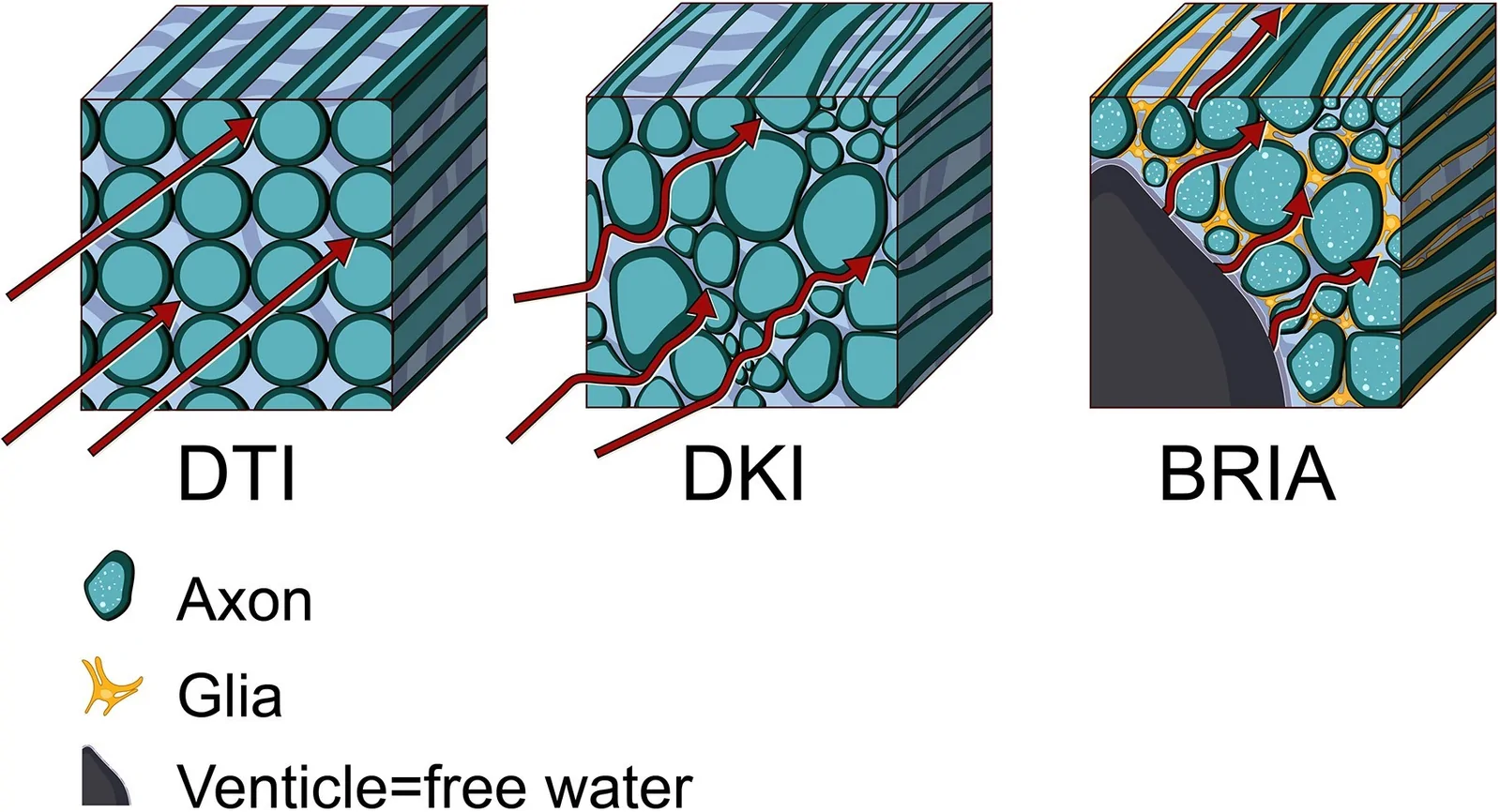
**Schematic illustration of diffusion approaches**. DTI likely oversimplifies white matter by assuming uniform, aligned axons. DKI accounts for non-Gaussian diffusion, while BRIA also separates three water pools and applies Bayesian fitting. Together, these approaches provide progressively more detailed insight into white matter microstructure. DTI, diffusion tensor imaging; DKI, diffusional kurtosis imaging; BRIA, Bayesian rotation invariant approach.

To characterize underlying WM microstructure, we applied a biophysical model incorporating three water compartments: intra-axonal, extra-axonal and free water fractions to account for partial volume effects. Given the known degeneracy of this standard WM model, we incorporated prior information on the plausible distribution of the involved diffusion parameters to stabilize the scalar parameter's estimation. The numerical solution of the standard diffusion model^9,26^ was based on BRIA approach that leverage Bayesian inference and multi-shell diffusion-weighted imaging data to robustly estimate parameters while accounting for noise and model complexity. Parameter estimation was performed using the publicly available Matlab implementation of BRIA provided by the methods developers.^27^

The combination of scalar intra- and extra-axonal metrics, along with rotational invariance, allows for a more detailed and less orientation-dependent characterization of WT microstructure compared to conventional DTI.^10^ The resulting metrics were accompanied by microDTI parameters analogous to conventional DTI scalar metrics, estimated from the intra- and extra-axonal diffusivities (D_IAW_ and D_EAW_) and their corresponding water fractions (V_IAW_ and V_EAW_).

### Quality control

All diffusion data passed the quality control check using YTTRIUM algorithm.^28^ In brief, this method applies a clustering approach to 2D data integrating mean skeleton diffusion metrics with structural similarity measures between each individual diffusion image and the derived mean image. This procedure allowed us to evaluate the general data validity in terms of normative modelling.^29^

### Statistical analysis

We compared differences between groups using independent *t*-test for continuous variables and *χ*² test or Fischer's exact test for categorical variables. These analyses were performed using Stata version 19 (StataCorp, College Station, TX, USA). WM metrics across major tracts were analysed using tract-based spatial statistics (TBSS). The TBSS analysis was based on a template derived from the original study. To enhance the generalisability and facilitate cross-study comparison, we aligned the study derived template to the Developing Human Connectome Project (dHCP) 40 weeks reference atlas.^2^ This allows a detailed parcellation of brain regions, as a complementary source of information for comparing the groups. The adapted scheme of TBSS analysis is summarized in Fig. 3. In short, we selected five subjects with the highest image quality, free from artefacts or distortions and with a Kidokoro score of 0 (no pathology). These five images enabled us to create the FA template, which was aligned to the dHCP atlas and used as a reference image in TBSS pipeline.

**Figure 3:**
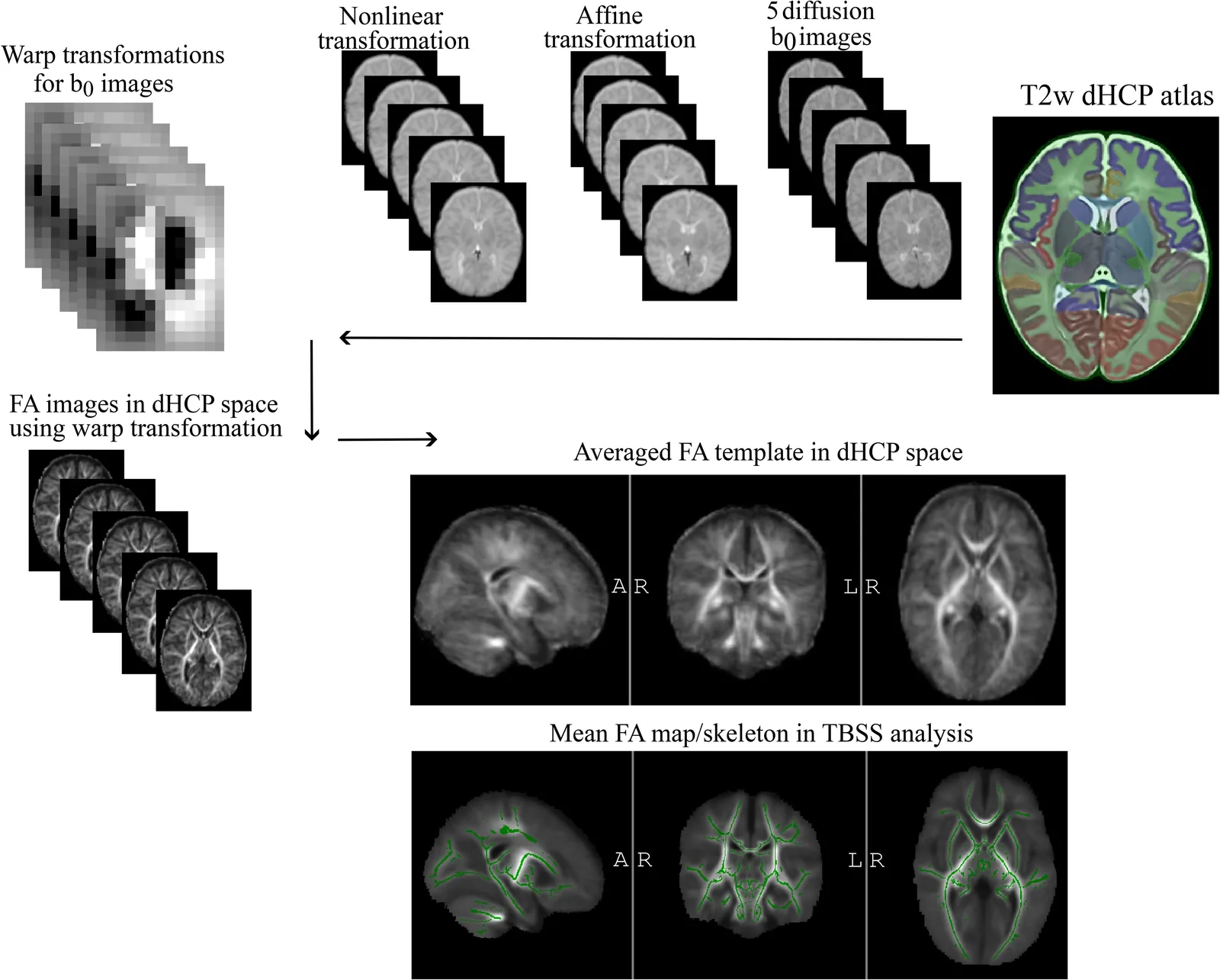
**Workflow scheme of FA template derivation for TBSS analysis**. Five healthy preterm infants with no visible brain abnormalities and Kidokoro scores of 0 were selected to generate a study-specific FA template. Individual FA maps were averaged to create the template which was then aligned to the dHCP atlas. Mean FA values were projected onto the common white matter skeleton to generate the final FA skeleton used for TBSS analysis. T2w, T2-weighted, FA, fractional anisotropy; TBSS, tract-based spatial statistics; dHCP, Developing Human Connectome Project.

The obtained study based mean FA skeleton was thresholded at FA > 0.15, and individual FA metrics were projected onto the skeleton prior to statistical analysis using randomize utility.^30^ Hereafter, all diffusion metrics were projected on the FA skeleton as well using *tbss_non_FA* utility from the FSL package. Following the family-wise error rate and threshold-free cluster enhancement, which was applied for 5000 permutations, significance was set at *P* < 0.05. Results were visualized as a colour-coded overlay on the mean FA skeleton. Since GA at birth, number of patients with suspected early-onset septicaemia and post-natal steroid exposure were unevenly distributed between the groups, as shown in Table 1, these variables were added as covariates to the general linear model.^18,19^

**Table 1:** Baseline demographics and neonatal characteristics

	Total ImNuT cohort		DTI-advanced group		*P*-value
	Control (*n* = 60)	ARA:DHA (*n* = 60)	Control (*n* = 31)	ARA:DHA (*n* = 33)	
Infants					
GA, weeks	26.2 (1.63)	26.6 (1.72)	**26.1** (**1.58)**	**26.9** (**1.39)**	**0**.**04**
Birth weight, g	833 (255)	879 (241)	839 (246)	904 (257)	0.31
Weight *Z*-score	−1.25 (1.35)	−1.22 (1.46)	−1.03 (1.22)	−1.38 (1.76)	0.37
Birth HC, cm	23.9 (2.42)	24.4 (2.05)	24.0 (2.55)	24.7 (1.99)	0.17
HC *Z*-score HC	−0.68 (0.93)	−0.64 (0.95)	−0.56 (1.00)	−0.60 (1.00)	0.87
Birth length^b^, cm	33.5 (3.49)	33.7 (2.98)	33.6 (3.56)	34.0 (3.01)	0.67
Length *Z*-score	−1.66 (1.87)	−1.86 (1.73)	−1.37 (1,87)	−2.04 (1.90)	0.16
PMA at scan			40.9 (1.00)	41.2 (1.22)	0.34
Baseline characteristics					
Small for GA	12/60 (20.0)	11/60 (18.3)	5/31 (16.1)	7/33 (21.2)	0.60
Male sex	31/60 (51.7)	35/60 (58.3)	17/31 (54.8)	20/33 (60.6)	0.64
Plurality					
Single	47/60 (78.3)	44/60 (73.3)	24/31 (77.4)	25/33 (75.7)	0.88
Twin	10/60 (16.7)	16/60 (26.7)	7/31 (22.6)	8/33 (24.2)	
Triplet	3/60 (5.0)				
5′APGAR score < 5	9/60 (15.0)	3/60 (5.0)	4/31 (12.9)	3/33 (9.1)	0.63
Early-onset septicaemia^a^	3/60 (5.0)	11/60 (18.3)	**0/31 (0)**	**7/33 (21.2)**	**0**.**01**
MV > 7 days	30/60 (50.0)	22/60 (36.7)	17/31 (54.8)	10/33 (30.3)	0.05
Post-natal steroids^b^	28/60 (46.7)	21/60 (35.0)	**17/31 (54.8)**	**9/33 (27.0)**	**0**.**03**
ARA, mol%	13.0 (3.5)	13.4 (4.5)	14.1 (3.35)	14.2 (4.37)	0.87
DHA, mol%	2.56 (0.83)	2.51 (0.76)	2.37 (0.73)	2.52 (0.83)	0.57
Neonatal morbidities^c^					
No IVH	48/60 (80.0)	47/60 (78.3)	24/31 (77.4)	27/33 (81.8)	0.66
IVH grade III–IV	4/60 (6.7)	7/60 (11.7)	1/31 (3.2)	3/33 (9.1)	0.94
PVL ≥ stage III	1/60 (1.7)	-	-	-	
No ROP	32/55 (58.2)	38/55 (69.1)	14/31 (45.2)	23/33 (69.7)	0.05
ROP stage ≥ III	7/55 (12.7)	3/55 (5.4)	6/31 (19.4)	2/33 (6.1)	0.10
NEC stage ≥ II	2/60 (8.3)	2/60 (3.3)	1/31 (3.2)	0/33 (0.0)	0.30
BPD	30/55 (54.5)	23/55 (41.8)	18/31 (58.1)	14/33 (45.2)	0.21
PDA	41/58 (70.7)	37/57 (64.9)	24/31 (77.4)	21/33 (63.6)	0.23
Late-onset septicaemia	27/57 (47.4)	23/57 (40.4)	15/31 (48.4)	11/33 (33.3)	0.22
Major morbidity^d^	32/55 (58.2)	26/55 (47.3)	18/31 (58.1)	15/33 (45.5)	0.31

Values are presented as *n* (%) or mean (SD), and statistically significant differences (*P* < 0.05) are highlighted in bold.

BPD, bronchopulmonary dysplasia; GA gestational age; HC head circumference; IVL, intraventricular haemorrhage; MV, mechanical ventilation; NEC, necrotizing enterocolitis; PDA, patent ductus arteriosus; PMA, postmenstrual age; PVL, periventricular leukomalacia; ROP, retinopathy of prematurity.

^a^Early-onset septicaemia was defined as positive blood culture or clinical signs of infection with CRP > 10 pg/mL within 72 h after birth.

^b^Any dose.

^c^Data are missing for infants who died or were withdrawn.

^d^Major morbidity included BPD, severe brain injury (IVH grade III–IV or cystic PVL) or ROP stage ≥ 3.

## Results

In total, 121 infants born between April 2018 to January 2021 underwent randomization.^8^ Advanced diffusion metrics were available for 64 subjects, 33 in the ARA:DHA group and 31 in the control group. Details regarding study inclusion are presented in Fig. 1. Baseline demographics and neonatal characteristics are presented in Table 1, nutrient intakes in Supplementary Table 2 and growth in Supplementary Table 3. Reported adverse events and serious adverse events were similar between the groups and have previously been published.^31^ The two groups differed in the following parameters: GA at birth, post-natal steroid exposure and early-onset septicaemia (Table 1). Significant differences in TBSS analysis based on the kurtosis-derived DTI metrics are presented in Fig. 4.

**Figure 4:**
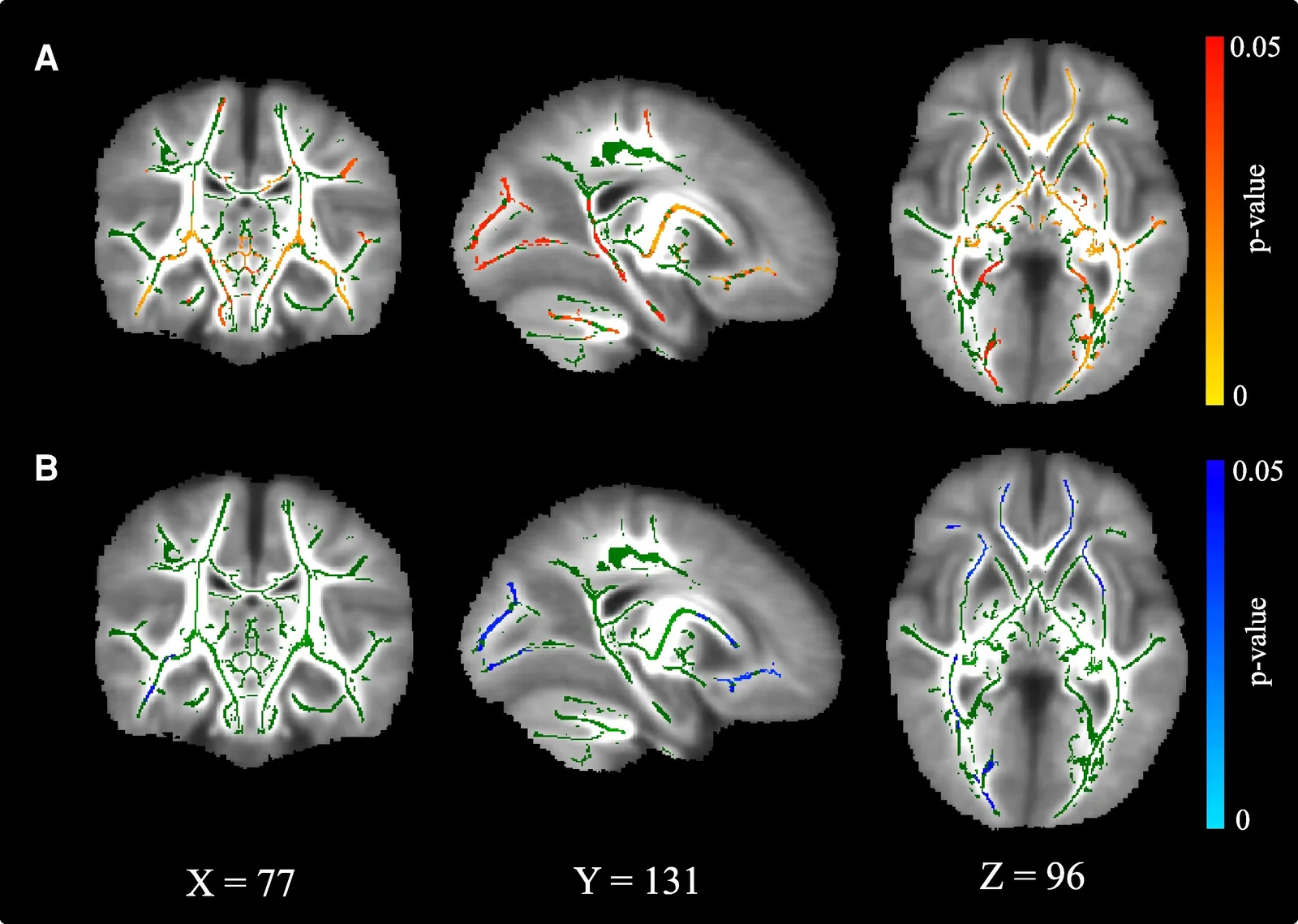
**The results of TBSS analysis for DTI metrics after application of diffusion-kurtosis framework**. **A** represents the KFA map and **B** represents the KRD-map. White matter skeleton from the TBSS analysis is presented in green and overlaid on mean KFA map (**A**) with colour coding showing areas with significant higher KFA (red to yellow), and **B** illustrates lower KRD (dark to light blue) in the ARA:DHA group (*n* = 33) compared to the control group (*n* = 31). Colour bars show the coding of significant *P*-values on the skeleton. Coordinates for dHCP atlas are presented as well. TBSS, tract-based spatial statistics; DTI, diffusion tensor imaging; KFA, kurtosis-derived fractional anisotropy, KRD, kurtosis-derived radial diffusivity; dHCP, Developing Human Connectome Project.

Kurtosis-derived FA (KFA) was higher in several major WM tracts in a diffusely spread manner in the ARA:DHA group with particularly pronounced increases in the posterior limb of internal capsule (PLIC)/cortico-spinal tracts and optic radiations. Differences in kurtosis-derived RD (KRD) were less marked with lower KRD values observed in the ARA:DHA group primarily in optic radiations and frontal tracts. MD and AD diffusion metrics did not show any significant differences. There were no significant differences in the DKI metrics between the groups (data not presented). Using the BRIA model, we identified significant group differences in microDTI metrics. Infants in the ARA:DHA group exhibited higher values of the following metrics: microFA, extra axonal water fraction (V_EAW_) and extra-axonal axial diffusivity (D_EAW_) and lower values of the following: microMD, microRD, microAD and free-water fraction (V_CSF_). The differences between groups appeared widespread but were most pronounced in major WM tracts that myelinate early and overlapping areas. The changes in microMD, D_EAW_ and V_CSF_ were asymmetrical with considerably more changes in the right hemisphere. All findings on BRIA analyses are summarized in Fig. 5. Absolute numbers (means) for the significant metrics in DTI and BRIA across the different brain regions are summarized in Supplementary Table 1.

**Figure 5:**
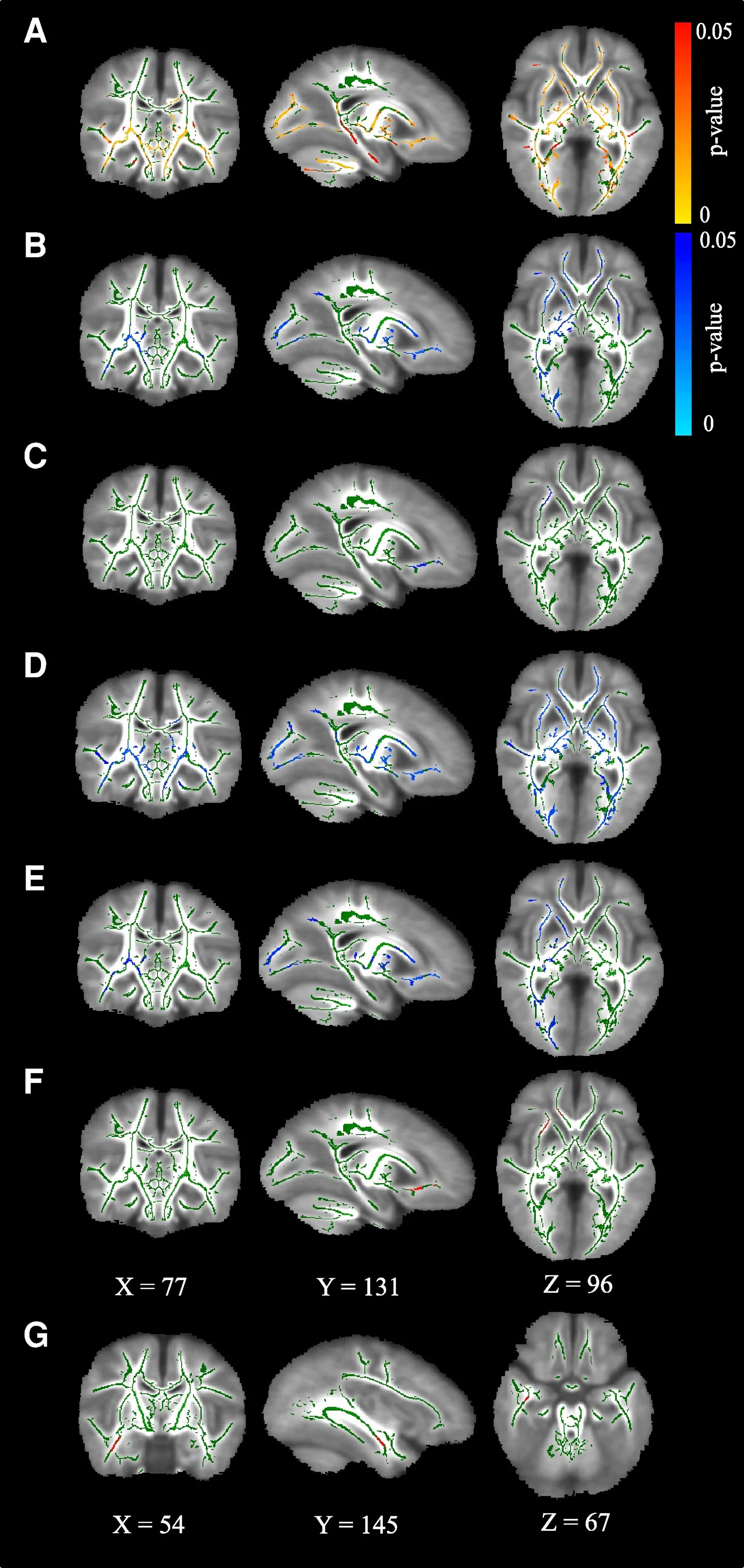
**The results of TBSS analysis for BRIA model**: (**A**) microFA; (**B**) microMD; (**C**) microAD; (**D**) microRD; (**E**) V_CSF_; (**F**) V_EAW_; (**G**) D_EAW_. The significant differences were found in microDTI metrics (microFA, microMD, microAD and microRD), in water fractions for extra-axonal (V_EAW_) and free-water (V_CSF_), and for extra-axonal axial diffusivity (D_EAW_). Colour bars show the coding of significant *P*-values on the skeleton as the following: red-yellow colour exhibits the significantly higher values in the ARA:DHA group (*n* = 33), and the blue-light blue colour exhibits the significantly higher metrics in the control group (*n* = 31).

## Discussion

In line with findings from the previously published results on a larger patient group from the ImNuT study,^8^ kurtosis-derived DTI metrics of this subgroup showed a significantly higher KFA and lower KRD in large overlapping WM areas, including tracts that usually myelinate early (e.g. PLIC and optic radiations). Increased KFA in WM likely reflects tissue reorganization, with higher axonal density and more parallel axon alignment in tract bundles, which restricts water diffusion perpendicular to the axons.^24^ The combination of increasing KFA and decreasing KRD suggest increased myelination of the axons in the ARA:DHA group compared to the control group. Importantly, application of the BRIA approach extends these findings: higher microFA values in major WM tracts in the ARA:DHA group and lower microRD in the same areas. Additionally, microMD and microAD were lower in the ARA:DHA supplemented group, further supporting the hypothesis of more advanced microstructural development. Together, the concordance between kurtosis-derived DTI and BRIA findings supports the robustness of the previously reported effects, while the advanced diffusion modelling applied here provides new insight into the underlying tissue organization that could not be resolved using DTI alone. The findings of higher extra-axonal water fraction (V_EAW_) and diffusivity (D_EAW_) in the ARA:DHA group were limited in extent and therefore difficult to interpret.

DKI metrics are sensitive to microstructural alterations associated with inflammation and oedema.^32^ In neonatal animal models of hypoxic-ischaemic encephalopathy, DKI detects injury-related changes within hours of the hypoxic-ischaemic insult with decreasing kurtosis metrics corresponding with neuronal swelling and inflammatory infiltration.^33,34^ Human neonatal studies similarly show that decreasing kurtosis can distinguish severity of WT injury, offering potential prognostic value in infants with encephalopathy.^35^ The absence of significant differences in kurtosis metrics in our study suggests that the observed changes in microFA, microRD and V_CSF_ are unlikely to be driven by inflammatory processes. Rather, they are more consistent with differences in normal developmental processes, such as more densely packed axons, extracellular reorganization and emerging myelination. The anatomical distribution of the significant changes further supports this interpretation.

The rightward asymmetry in microMD, D_EAW_ and V_CSF_ may similarly reflect a normal maturational process. Results from earlier studies regarding asymmetric maturation in the preterm have been conflicting, especially when it comes to studies on WT.^36^ The right dominance can however be interpreted in line with the right hemisphere conservatism theory, stating that the right hemisphere develops earlier and that its development is less subjective to external influences.^37^

Standard DTI metrics can be disproportionately affected by low coherence and reduced anisotropy, complicating histological translation. The obtained results allow detection and localization of brain tissue changes at a finer scale, incorporating metrics that are less dependent on fibre orientation and more robust than conventional DTI parameters. Other studies providing compartment-specific characteristics of the WM microstructure, such as Neurite Orientation Dispersion and Density Imaging (NODDI)^38,39^ have shown correlations between diffusion metrics and WM connectivity as well as cognitive and behavioural functions.^40^ However, NODDI relies on fixed diffusivity assumptions that can lead to parameter bias when applied to the neonatal brain.^41,42^ BRIA addresses these limitations by adopting a Bayesian framework that estimates distributions of diffusion parameters rather than fixing them *a priori* and by using rotationally invariant metrics that reduce dependence on fibre orientation modelling.

Altogether, we believe that advanced diffusion metrics may be more sensitive for detecting microstructural WT alterations that could have clinical implication in this population.

### Strengths and limitations

The main strength of this study is its double-blind randomized controlled design where all the participants followed a standardized nutrition protocol, combined with a very advanced and rigid scan protocol. We also obtained a comprehensive assessment of nutrient intakes and growth. In this subgroup analysis, there were significant differences between the groups in GA, early-onset septicaemia and post-natal steroid exposure. To address these imbalances and reduce confounding, these variables were incorporated as covariates in the general linear model. Another strength is the application of three different diffusion approaches providing more detailed information regarding the brain microstructure.

The nature of this study does however not enable a clear translation to an underlying biophysical mechanism of the observed effects, in other words whether ARA:DHA supplementation serves as a neuroprotective agent or actively enhances the myelination process in these infants. This could possibly be clarified using higher diffusion weightings or more sophisticated diffusion protocols such as *b-*tensor encoding.^43^ In addition, multi-echo time pulse sequences could reveal changes in myelin water fraction, which is a robust proxy for myelination. However, such sequences are typically time-consuming and often require substantial compromises in protocol development.

This study is also limited by its relatively small sample size due to the quality requirements of the scans for analysis. Initial power calculations were based on conventional DTI parameters rather than advanced diffusion metrics.^14^ We did not acquire baseline scans before supplementation, as this is not feasible or ethically justifiable in very preterm infants who are often clinically unstable and require respiratory and thermal support.

White matter development in very preterm infants is influenced by multiple inter-related factors, including GA, inflammation, respiratory morbidity and nutrition as illustrated in Supplementary Fig. 1. Although we adjusted for the clinical variables that differed and observed no group difference in human milk intake, residual confounding from unmeasured inflammatory exposures, such as histological chorioamnionitis, cannot be fully excluded. Another limitation is that our diffusion MRI assessments are restricted to WM, which constitutes a relatively small portion of the brain and is not fully developed at term-equivalent age. The infant brain consists of high-water content and low myelin, with myelination occurring asynchronously across brain regions. This results in heterogeneous diffusivity patterns complicating brain segmentation methods.^39^ Finally, the clinical implications of these MRI findings in the brain at term are unclear and need to be elucidated in clinical long-term follow-up studies which is also our intention in the ImNuT trial.

## Conclusion

Advanced diffusion approaches revealed microstructural changes in the intra- and extra-axonal WT water compartments of preterm infant brain following ARA and DHA supplementation.

## Supplementary Material

fcag362_Supplementary_Data

## Data Availability

Data presented in this manuscript is available from the corresponding author upon reasonable request but is not publicly available due to GDPR restrictions. Individual data sharing must be approved by the local ethics committee.
