# Supplementary material for: Arachidonic and docosahexaenoic acid supplementation affects white matter microstructure in preterm infants
Source: Brain Commun. 2026 Oct 11;8(5):fcag362. doi: 10.1093/braincomms/fcag362 (PMC13663797; doi:10.1093/braincomms/fcag362)
Supplement: Supplementary file 1 [file fcag362_supplementary_data.docx]

**Supplementary information for Arachidonic- and docosahexaenoic acid supplementation affects white matter microstructure in preterm infants**

**Supplementary Table 1: Differences in DTI and BRIA metrics across brain regions**

| **Regions** | **Temporal lobe** | **Occipital lobe** | **Frontal lobe** | **Pariental lobe** | **Cerebellum** | **Brainstem** | | **Corpus callosum** |
| --- | --- | --- | --- | --- | --- | --- | --- | --- |
|  | **FA, mean (SD)** | | | | | | | |
| **ARA:DHA group** | 0.206  (0.023) | 0.230  (0.023) | 0.218  (0.024) | 0.231  (0.024) | 0.231  (0.018) | 0.263  (0.017) | 0.362  (0.039) | |
| **Control group** | 0.199  (0.019) | 0.216  (0.019) | 0.206  (0.021) | 0.220  (0.019) | 0.226  (0.014) | 0.255  (0.014) | 0.334  (0.034) | |
|  | **RD µm^2^/ms, mean (SD)** | | | | | | | |
| **ARA:DHA group** | 1.299  (0.088) | 1.330  (0.086) | 1.318  (0.086) | 1.325  (0.083) | 0.980  (0.059) | 1.000 (0.034) | 1.248 (0.083) | |
| **Control group** | 1.317  (0.079) | 1.374  (0.103) | 1.347  (0.104) | 1.351  (0.088) | 0.978 (0.057) | 1.005 (0.039) | 1.300 (0.074) | |
|  | **MicroFA, mean (SD)** | | | | | | | |
| **ARA:DHA group** | 0.205 (0.025) | 0.206 (0.025) | 0.224 (0.031) | 0.227 (0.028) | 0.342 (0.028) | 0.329 (0.015) | 0.282 (0.024) | |
| **Control group** | 0.196 (0.023) | 0.193 (0.029) | 0.215 (0.036) | 0.219 (0.029) | 0.336 (0.030) | 0.319 (0.015) | 0.262 (0.029) | |
|  | **MicroRD µm^2^/ms, mean (SD)** | | | | | | | |
| **ARA:DHA group** | 1.434 (0.107) | 1.494 (0.108) | 1.499 (0.117) | 1.499 (0.111) | 0.952 (0.090) | 1.026 (0.048) | 1.472 (0.083) | |
| **Control group** | 1.476 (0.113) | 1.556 (0.142) | 1.543 (0.152) | 1.538 (0.127) | 0.964 (0.108) | 1.052 (0.077) | 1.538 (0.105) | |
|  | **MicroAD µm^2^/ms, mean (SD)** | | | | | | | |
| **ARA:DHA group** | 1.898 (0.080) | 1.987 (0.074) | 2.039 (0.059) | 2.053 (0.063) | 1.667 (0.108) | 1.731 (0.074) | 2.226 (0.041) | |
| **Control group** | 1.926 (0.088) | 2.027 (0.099) | 2.067 (0.088) | 2.076 (0.077) | 1.670 (0.132) | 1.739 (0.116) | 2.248 (0.057) | |
|  | **MicroMD µm^2^/ms, mean (SD**) | | | | | | | |
| **ARA:DHA group** | 1.589 (0.096) | 1.659 (0.096) | 1.679 (0.097) | 1.684 (0.094) | 1.190 (0.090) | 1.260 (0.053) | 1.723 (0.067) | |
| **Control group** | 1.626 (0.103) | 1.713 (0.127) | 1.717 (0.130) | 1.717 (0.110) | 1.199 (0.111) | 1.281 (0.088) | 1.774 (0.087) | |
|  | **Free water fraction (v_CSF_), mean (SD)** | | | | | | | |
| **ARA:DHA group** | 0.283 (0.050) | 0.318 (0.048) | 0.316 (0.050) | 0.322 (0.049) | 0.138 (0.035) | 0.166 (0.025) | 0.351 (0.034) | |
| **Control group** | 0.302 (0.054) | 0.346 (0.066) | 0.338 (0.068) | 0.340 (0.058) | 0.142 (0.049) | 0.175 (0.045) | 0.376 (0.048) | |
|  | **Extra-axonal water fraction (v_EAW_), mean (SD)** | | | | | | | |
| **ARA:DHA group** | 0.618 (0.056) | 0.573 (0.046) | 0.580 (0.045) | 0.569 (0.046) | 0.662 (0.061) | 0.665 (0.046) | 0.487 (0.031) | |
| **Control group** | 0.599 (0.059) | 0.545 (0.064) | 0.558 (0.062) | 0.551 (0.056) | 0.660 (0.078) | 0.657 (0.075) | 0.468 (0.049) | |
|  | **Extra-axonal axial diffusivity (D_EAW_) µm^2^/ms, mean (SD)** | | | | | | | |
| **ARA:DHA group** | 1.222 (0.044) | 1.251 (0.032) | 1.342 (0.049) | 1.343 (0.041) | 1.128 (0.039) | 1.192 (0.026) | 1.488 (0.030) | |
| **Control group** | 1.214 (0.038) | 1.235 (0.032) | 1.325 (0.048) | 1.330 (0.038) | 1.125 (0.043) | 1.186 (0.032) | 1.468 (0.039) | |

**Supplementary Table 2**: Nutrient intake during neonatal hospitalisation^a^

|  | **ARA:DHA**  (n= 33) | **Control**  (n = 31) | **p-value** | ESPGHAN 2022^b^ |
| --- | --- | --- | --- | --- |
| Any human milk, ml/kg | 140.7 (18.9) | 138.3 (18.0) | 0.60 | 150-180 |
| Energy, kcal/kg | 136.4 (8.1) | 135.0 (8.8) | 0.51 | 115-140 |
| Protein, g/kg | 4.2 (0.3) | 3.9 (0.3) | **<0.01** | 3.5-4.0 |
| Carbohydrate, g/kg | 12.9 (0.8) | 12.7 (0.7) | 0.38 | 11-15 |
| Fat g/kg | 7.2 (0.6) | 7.2 (0.7) | 1 | 4.8-8.1 |

Values are means (±SD) and statistical differences are highlighted in bold.

^a)^Only infants with complete nutritional data were included in the analysis.

^b)^ESPGHAN (European Society for Paediatric Gastroenterology, Hepatology and Nutrition)

**Supplementary Table 3**: Infant characteristics of the groups at MRI-acquisition^a^.

|  | **ARA:DHA (n=33)** | **Control (n=31)** | **p-value** |
| --- | --- | --- | --- |
| Weight at MRI, g | 3510.6 (530.3) | 3542.0 (372.2) | 0.79 |
| Δz-score Weight | 0.4 (1.14) | 0.3 (0.89) | 0.73 |
| HC at MRI, cm | 36.3 (1.39) | 35.6 (1.28) | **0.03** |
| Δz-score HC | 0.8 (0.93) | 0.3 (0.94) | **0.03** |
| Length at MRI, cm | 50.6 (2.42) | 49.5 (2.05) | 0.06 |
| Δz-score Length | 0.8 (1.43) | -0.4 (1.82) | **0.01** |

Values are means (±SD) and statistical differences are highlighted in bold.

^a)^Only infants with complete nutritional data were included in the analysis.

**Supplementary Figure 1.** Direct acyclic graph (DAG) illustrating hypothesised causal relationships between arachidonic and docosahexaenoic acid supplementation and white matter maturation. Human milk intake and histological chorioamnionitis are possible confounders, while growth parameters and inflammation are possible mediators.
